# Intestinal mucosal immune responses induced by oral administration of chitosan nanoparticles encapsulating the PEDV S1 protein

**DOI:** 10.1186/s13567-025-01695-6

**Published:** 2025-12-19

**Authors:** Kai Su, Jing Ren, Yanan Zhang, Chen Yuan, Yawen Wang, Liu Yang, Lizhi Fu, Tingli Fan, Qinye Song

**Affiliations:** 1 College of Veterinary Medicine, Hebei Agricultural University, Baoding, 071000 China; 2 Hebei Veterinary Biotechnology Innovation Center, Baoding, 071000 China; 3 National Center of Technology Innovation for Pigs, Chongqing, 400000 China; 4 Department of Agricultural and Animal Husbandry Engineering, Cangzhou Technical College, Cangzhou, 061001 China

**Keywords:** PEDV, S1 protein, chitosan nanoparticles, oral administration, mucosal immunity, piglet

## Abstract

Porcine epidemic diarrhea (PED), a highly contagious intestinal disease caused by PEDV, threatens newborn piglets and causes enormous losses in the swine industry. Intestinal mucosal immunity is vital for defense against PEDV. Chitosan (CS), a mucin-adhesive biodegradable polysaccharide, has emerged as a promising mucosal vaccine delivery vector and antigen adjuvant. To develop a PEDV mucosal vaccine, we prepared S1-CS nanoparticles (NPs) loaded with the S1 domain of the PEDV spike (S) protein. We evaluated their ability to induce mucosal immune responses in mice via different inoculation routes and to confer immune protection in piglets after oral inoculation. The results showed that S1-CS NPs exhibited no cytotoxicity and remained stable in simulated gastric fluid in vitro. Compared with oral administration, intramuscular inoculation of S1-CS NPs induced higher levels of PEDV-specific serum IgG in mice. However, PEDV-specific IgA was detected only in the serum and intestinal lavage fluid of orally vaccinated mice. Oral administration also elicited higher IFN-γ levels than intramuscular injection, suggesting stronger activation of cellular immunity. In piglets, oral S1-CS NPs induced serum neutralizing antibodies and IgA responses in both serum and intestinal mucosa, increased the number of IgA-secreting cells in the intestine, reduced viremia and virus shedding, and improved intestinal villus morphology. These findings indicate that the S1-CS NPs developed in this study represent promising candidates for an oral mucosal PEDV vaccine.

## Introduction

Porcine epidemic diarrhea virus (PEDV) is a member of the genus *Alphacoronavirus* within the family *Coronaviridae*. PEDV causes acute diarrhoea and dehydration, accompanied by viremia, which leads to high mortality in neonatal piglets [1]. The classical genetic group I (GI) strain was initially discovered in Europe in the 1970s. However, since the emergence of the epidemic GⅡ strain of PEDV in China in 2010, the prevalence of PEDV GII strains has increased widely in Asia and North America [2]. The GⅡ strains of PEDV exhibit increased virulence, resulting in considerable economic losses in the global swine industry [3].The spike (S) protein, a type I glycoprotein trimer consisting of S1 and S2 subunits situated on the surface of PEDV, plays a pivotal role in viral infection among all structural proteins of the virus. The S1 subunit is responsible for receptor binding to porcine intestinal epithelial cells, whereas the S2 subunit mediates the fusion of the virus with the host cell membrane, releasing the viral genome into the cytoplasm for replication [4]. Notably, the binding of S1 to the porcine aminopeptidase (pAPN) receptor contributes to enhanced mucosal immune responses [5]. As the primary glycoprotein, the S1 region of the S protein is a major target for the development of new vaccine candidates [6–8]. Antigens prepared on the basis of the S1 protein can effectively induce protective immune responses [7].

For some viruses, such as PEDV, that infect and replicate in the mucosa, vaccine design must prioritize the induction of mucosal immunity to protect local mucosal tissues. Moreover, given that PEDV poses the greatest threat to neonatal piglets and that their immune systems are not yet fully mature, the acquisition of passive immunity through milk is the sole pathway through which piglets obtain maternal immune protection [9]. The survival rate of infected newborn piglets is positively correlated with IgA antibodies in sow milk [10]. Traditional PEDV vaccines cannot effectively induce intestinal mucosal immunity, let alone passive immunity acquired by newborn piglets through maternal milk. The gastrointestinal tract has the largest mucosal tissue and intestinal immune system, comprising 70% of lymphocytes in the body and featuring both complete inductive and effector sites [11]. Typically, the gastrointestinal immune system is constantly challenged with antigens from the lumen [12].

Particulate chitosan systems protect antigens from degradation and enhance the uptake of the particles by antigen-presenting cells (APCs), macrophages and M cells at mucosal sites and other sites of administration [13]. Chitosan is a naturally derived linear polysaccharide formed by partial deacetylation of chitin. As a biodegradable and biocompatible cationic polymer, chitosan has a wide range of applications in biomedical fields, such as in the preparation of gels, pastes, tablets, microspheres, and nanoparticles as drug or vaccine delivery carriers [14]. Owing to their adhesive properties, encapsulating antigens in chitosan nanoparticles and administering them to local mucosal tissues can further prolong the antigen residence time, strengthening the interaction between antigens and immune cells [15].

**Figure 1:**
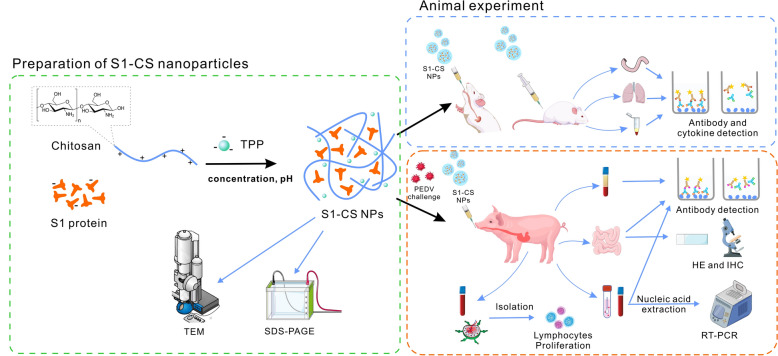
**Graphical abstract**. Schematic diagram depicting the preparation of S1-CS nanoparticles, animal immunization, and sample collection and measurement. S1-CS nanoparticles were prepared through ionic crosslinking by optimizing the proportions and conditions of the necessary materials. The immune effects of S1-CS NPs were evaluated in mice and piglets by intramuscular or oral administration.

In this study, the PEDV S1 protein was encapsulated in chitosan nanoparticles, and intramuscular and oral immunizations were performed in mice to evaluate the mucosal immune responses induced by S1-chitosan nanoparticles (S1-CS NPs). Subsequently, the immune effects of S1-CS NPs were assessed in piglets following oral immunization (Figure 1), providing new insights for the development of PEDV mucosal vaccines.

## Materials and methods

### Cells and virus

The African green monkey kidney epithelial (Vero)-CCL81 cell line from the ATCC and the PEDV QY-2016 strain (GenBank ID: MH244927) were preserved in our laboratory. The Vero-CCL81 cells were propagated in Dulbecco’s modified Eagle’s medium (DMEM; Gibco, California, USA) supplemented with 10% FBS (Sigma Aldrich, USA), 100 IU/mL penicillin, and 100 μg/mL streptomycin (Solarbio, Beijing, China) in a humidified incubator at 37 °C and 5% CO_2_. The PEDV QY-2016 strain was cultured in Vero-CCL81 cells in DMEM supplemented with 10 µg/mL trypsin (Solarbio), 0.3% tryptose phosphate broth (Hope Bio, China), 100 IU/mL penicillin, and 100 μg/mL streptomycin (Solarbio). The TCID_50_ of the virus was calculated according to the Reed‒Muench two-equation method.

### Preparation of S1-CS NPs

The preparation of the PEDV S1 protein was performed according to our previous work[16]. Chitosan (CS, deacetylated ≥ 95%, viscosity 100–200 mpa.s) and sodium tripolyphosphate (TPP, analytical grade) were purchased from Shanghai Aladdin Biochemical Technology Co., Ltd. To determine the optimal encapsulation efficiency (EE) and loading efficiency (LE) of chitosan for proteins, the encapsulation of S1 protein within chitosan was implemented. Briefly, S1 protein was added to 0.1% (w/v) CS solutions with varying pH values ranging from 4.2–6.0, after which the mixtures were centrifuged at 12 000 r/min for 10 min. Then, the protein concentration in the supernatant was determined via a BCA protein assay kit. The amount of precipitated protein was calculated before cross-linking to select the optimal reaction pH. The amount of S1 protein added to each reaction system (with 10 mg of CS) was 1.0 mg, 2.0 mg, 3.0 mg, or 4.0 mg, with 3 replicates for each group, aiming to achieve a higher EE and minimize antigen waste. Moreover, 1.7 mL or 2.8 mL of 0.1% (w/v) TPP was added to ensure full crosslinking of the CS according to the emulsion morphology. Following centrifugation at 12 000 r/min for 10 min, the supernatants were collected separately to measure the protein concentration, and the precipitates were the S1-CS nanoparticles. The calculation formulas for EE and LE are as follows:

$$\mathrm{EE}=\frac{\mathrm{TP}-\mathrm{FP}}{\mathrm{TP}}\times 100\mathrm{\%}$$

where TP is the total protein/(mg) and FP is the amount of free protein/(mg).

$$\mathrm{LE}=\frac{\mathrm{EP}}{\mathrm{EP}+\text{Carrier content}}\times 100\mathrm{\%}$$

where EP is the encapsulated protein/(mg), and the carrier content is the mass of CS/(mg).

### Analysis of the S1 protein in S1-CS NPs

To estimate S1 protein in S1-CS NPs, SDS‒PAGE was performed with different concentrations of S1 protein as the standard, and a standard curve was drawn according to the protein band grayscale measured by ImageJ processing software. After crosslinking, the S1-CS NPs were collected by centrifugation at 12 000 r/min for 30 min at 4 °C and washed repeatedly with deionized water by pipetting and aspirating. The S1-CS NPs were subsequently resuspended in deionized water for SDS‒PAGE and quantitative analysis on the basis of the standard curve.

### Transmission electron microscopy

The S1-CS NP suspension was dropped onto copper grids and vacuum-dried to obtain the samples. The NPs were observed under a transmission electron microscope (TEM) (JEM1400, JEOL, Tokyo, Japan), which was operated at 80 kV. Digital images were acquired with an AMT camera.

### Cytotoxicity assay of S1-CS NPs

A CCK-8 (Sopo Biotech, Guangzhou, China) assay was performed to evaluate the cytotoxicity of the S1 protein, CS and S1-CS NPs in vitro. PK-15 cells in the logarithmic growth phase were maintained in 1640 medium (Wisent, Quebec, Canada) supplemented with 10% FBS (Wisent). A total of 5 × 10^4^ PK-15 cells was placed in a 96-well plate and incubated at 37 °C with 5% CO_2_ for 24 h. The supernatant was discarded, and the cells were gently washed three times with PBS. Various final concentrations (1, 10, 100, 250, and 500 μg/mL) of the CS, S1 protein, and S1-CS NPs were added to each well. Cell control and blank wells were set up. After incubation for 24 h, 10 μL of CCK-8 solution was added to each well, and the cells were incubated for an additional 3 h, with 3 replicates for each group. The absorbance at 562 nm was measured via a spectrophotometer (Biotech Synergy HTX, Vermont, USA), and the cytotoxicity of the CS, S1 protein, and S1-CS NPs was evaluated according to the relative survival rate (%) of the cells via the following formula:

$$\text{Cell Relative Survival Rate }\left(\mathrm{\%}\right)=\frac{\mathrm{OD}\left(\mathrm{treat}\right)-\mathrm{OD}\left(\mathrm{blank}\right)}{\mathrm{OD}\left(\text{Cell control}\right)-\mathrm{OD}\left(\mathrm{blank}\right)}\times 100\mathrm{\%}$$

### In vitro simulated digestion assay

To investigate the resistance of S1-CS NPs to gastric degradation/digestion, S1-CS NPs containing 600 μg of S1 protein were mixed with 9 mL of pH 1.2 simulated gastric fluid containing 3.2 mg/mL pepsin (Bioduly, Nanjing, China) and incubated for 0, 10, 20, 30, 60, 90, 120, 150, or 180 min at 37 °C. Controls of S1 protein and CS (1 mg/mL) were established. Each treatment was performed with 3 replicates. After centrifugation, 50 μL of the supernatant was removed from the mixed mixture for S1 protein concentration analysis by enzyme-linked immunosorbent assay (ELISA). Briefly, 96-well ELISA plates (JET Biofil, Guangzhou, China) were coated with 100 μL of the collected samples at 37 °C for 1 h and then incubated overnight at 4 °C. After being washed with PBST (0.05% Tween-20 in phosphate-buffered saline (PBS), pH 7.4) 3 times, the plates were blocked with blocking buffer (5% skim milk powder in PBST) at 37 °C for 1 h. A total of 100 μL of mouse polyclonal antibody against the PEDV-S1 protein (diluted 1:300) was added to the wells and incubated at 37 °C for 1 h. After three washes, 100 μL of HRP-conjugated goat anti-mouse IgG (1:15000, Biodragon Tech, Beijing, China) was added and incubated at 37 °C for 1 h. Following three washes, 100 μL/well of tetramethylbenzidine (TMB) single-component substrate solution (Solarbio) was added and incubated in the dark at room temperature (RT) for 15 min. Finally, 50 μL of stop solution and 2 mol/L sulfuric acid were added to each well to terminate the reaction, and the OD values at 450 nm for each well were measured via a multimode microplate reader (Biotech Synergy HTX, USA).

### Animal immunization and sample collection

To evaluate the immunogenicity of S1-CS NPs, systemic and mucosal immune responses induced by oral and intramuscular S1-CS NPs were compared in mice, and the immunoprotective effects of S1-CS NPs on piglets were subsequently evaluated via oral immunization and challenge tests.

1. Experiment in mice: S1 protein was mixed with an equal volume of IMS1313 water adjuvant (Seppic, Paris, France), and the S1-CS NPs were dissolved in sterilized deionized water at 4 °C for immunization. Twenty-four specific-pathogen-free (SPF) female BALB/c mice (Changsheng Biotech, Liaoning, China) aged 6–8 weeks were randomly divided into 4 groups, the S1/IM group, the S1-CS/IM group, the S1-CS/IO group, and the blank group, with 6 mice per group. The mice in the S1/IM and S1-CS/IM groups were immunized intramuscularly (IM) with a dose of 40 μg of S1 protein, whereas the mice in the S1-CS/IO group were orally immunized via a gavage needle with S1-CS NPs containing 40 μg of S1 protein. All the treatment groups were immunized 3 times at 2 week intervals. The mice in the blank group were not immunized. Blood samples were collected from the tail vein of the mice before and weekly after immunization, and the serum samples were separated and stored at −20 °C for detection of antibodies and cytokines. Fourteen days after the second immunization and 21 days after the third immunization, 3 mice from each group were anaesthetized by intramuscular injection of Su-mian-xin II (0.1 mL/20 g body weight). The intestinal contents and bronchoalveolar lavage fluid were collected for the detection of specific secretory IgA. Briefly, small intestinal segments from the mice were cut longitudinally, and the intestinal lumen was repeatedly rinsed with 4 mL of precooled PBS. The rinsing fluid was collected and centrifuged at 8000 r/min for 10 min at 4 ℃, and the supernatant was collected for ELISA detection of mucosal IgA. Moreover, a small incision was made in the middle of the trachea, and a blunt needle was inserted and fixed to prevent leakage. One millilitre of precooled PBS was slowly injected, and the mixture was transferred to a centrifuge tube in triplicate. The collected lavage fluid was centrifuged at 8000 r/min for 10 min at 4 ℃, and the supernatant was collected for detection.
2. Experiment with piglets: Fifteen 21 day-old piglets (comprising Duroc, Landrace, and Yorkshire breeds) were obtained from the Hebei Tangsheng industrialized pig farm. The piglets were free of PEDV and anti-PEDV antibodies according to the analysis of serum and rectal swabs by RT‒PCR and ELISA, respectively. Fifteen piglets housed in different isolation pens under similar rearing conditions were randomly divided into 3 groups (S1-CS, challenge control and blank groups), with 5 piglets in each group. Piglets in the S1-CS groups were orally inoculated with a needleless injector two times with 2 mL of S1-CS NPs containing 1000 μg of S1 protein at 2 week intervals. In the challenge control and blank groups, the piglets were administered PBS. At 14 days after the second immunization, each piglet, except those in the blank group, was challenged orally with 10 mL of PEDV containing 10^5.5^ TCID_50_. The experimental procedures used for piglet immunization, challenge and sample collection are shown in Figure 4A.

Blood samples collected from the anterior vena cava of piglets weekly before and after immunization, as well as saliva, nasal mucus and faecal samples for RT‒PCR and antibody detection, were stored at −20 °C. After challenge, the clinical manifestations were observed every day, and the shapes of the piglet feces were scored as follows: 0, solid; 1, pasty; 2, semiliquid; and 3, liquid. Serum, saliva, nasal mucus and fecal samples were collected at 0, 3, 7, and 14 days post-challenge (dpc) for RT‒PCR and antibody detection. On day 14 post-challenge, the piglets were euthanized by injecting 2 mL of chlorpromazine hydrochloride through the ear vein at 3 mg/kg BW, and macroscopic and microscopic lesions were detected in the small intestine. Approximately 20 cm segments from the mid-duodenum, mid-jejunum, and mid-ileum and their contents were collected for the detection of IgA antibody-secreting cells or mucosal IgA antibodies.

### ELISA for specific antibodies or cytokines

PEDV S1-specific IgG and IgA antibodies in mouse serum, intestine and bronchoalveolar lavage fluid and in piglet serum, saliva, nasal mucus and intestinal contents were measured by ELISA[16]. Briefly, 96-well ELISA plates (JET Biofil) were coated with the PEDV S1 protein (2 μg/well) at 37 °C for 60 min and incubated overnight at 4 °C. After being washed 3 times with PBST (0.05% Tween-20 in phosphate-buffered saline (PBS), pH 7.4), the plates were blocked with blocking buffer containing 5% skim milk powder in PBST at 37 °C for 1 h. One hundred microliters of serum (1:40–1:100 dilution for IgG and 1:10–1:20 dilution for IgA), intestine or bronchoalveolar lavage fluid (1:2 dilution) were added to the wells of the plate and incubated at 37 °C for 60 min. Simultaneously, positive, negative, and blank controls were set up. After washing, 100 μL of HRP-conjugated goat anti-mouse IgG (1:15 000, Biodragon Tech), goat anti-pig IgG (1:5000, Solarbio), or HRP-conjugated goat anti-mouse IgA (1:5000, Biodragon Tech) or goat anti-pig IgA (1:10 000; Abcam, Britain) was added at 37 °C for 60 min. After washing 3 times, 100 μL of TMB single-component substrate solution (Solarbio) was added per well, followed by incubation at RT for 15 min. Finally, the reaction was terminated using 50 μL/well 2 mol/L H_2_SO_4_, and the OD_450nm_ value was measured immediately using a Multimode Microplate reader (Biotek Synergy HTX, USA). Three parallel repeated tests were performed for each sample. The cut-off value was calculated as the mean OD of 30 negative controls plus two standard deviations (SDs).

The levels of IFN-γ, IL-2 and IL-4 in mouse serum were detected using mouse cytokine ELISA kits (Shanghai Enzyme-linked Biotech, China) on day 14 after the second immunization. The procedure was performed according to the manufacturer’s instructions and the literature [16].

### Microserum neutralization test

The microserum neutralization test (SNT) was conducted according to previously reported methods [17]. Briefly, heat-inactivated piglet serum was serially diluted twofold and incubated with PEDV at a multiplicity of infection (MOI) of 0.2 for 1 h at 37 °C. The serum‒virus mixtures in each dilution were transferred to confluent Vero CCL-81 cell monolayers in 96-well plates and incubated for 1.5 h at 37 °C, after which the PEDV control and blank control were set up. After the fluid in the wells was discarded, the cells were washed twice with prewarmed PBS, followed by the addition of prewarmed maintenance medium for incubation for 24 h at 37 °C and 5% CO_2_. The plates were subsequently washed and fixed in 3.7% formaldehyde for 30 min, permeabilized with 0.1% Triton X-100 for 5 min, washed and blocked with 5% fat-free dry milk in pH 9.5 PBS for 1 h at 37 °C. The cells were treated with an anti-PEDV N protein monoclonal antibody (A7) (from our lab) diluted 1:100 for 1 h at 37 °C, washed, and incubated with an HRP-conjugated goat anti-mouse IgG antibody (Biodragon Tech) diluted 1:10 000. The plates were washed with PBS and developed with TMB development solution (Solarbio). The absorbance was measured at 450 nm in a spectrophotometer (Biotek Synergy HTX) after the reaction was stopped by the addition of 2 M H_2_SO_4_. Two parallel repeated tests were performed for each sample. The percent inhibition of viral infection at each dilution of the serum was compared with that of the PEDV control. The 50% neutralization titre was defined as the reciprocal of the highest serum dilution for which 50% inhibition of infection was reached.

### Lymphocyte preparation

Peripheral blood mononuclear cells (PBMCs) were isolated from 10 mL of pig blood anticoagulant with heparin [18]. The lymphocytes from the Peyer's patches (PPs) were obtained from 20 cm of pig ileal tissue. The ileum was aseptically collected and immediately placed in ice-cold Hank’s solution (pH 7.2). After the contents and serosa of the tissues were removed, the ileal tissues were cut into 1 × 1 mm^2^ pieces and mixed in a preheated sterile dish with 10 mL of complex digestive enzyme in Hank’s solution containing 10% FBS, 1.5 mg/mL Dispase II (Sigma), 15 µg/mL DNase Ⅰ (Solarbio), and 2 × 10^4^ U/mL penicillin and streptomycin followed by shaking at 37 °C for 1 h. The digested ileal tissues were pressed through stainless steel 200-mesh screens with a cell collector (Solarbio) to obtain single-cell suspensions and washed 3 times with Hank’s solution. In addition, mesenteric lymph nodes (MLNs) were cut and digested as described above.

The lymphocytes were isolated via density gradient centrifugation via peripheral blood lymphocyte isolation solution (Solarbio) and suspended in RPMI 1640 medium (Wisent) supplemented with 10% FBS (Wisent), 100 IU/mL penicillin, and 100 μg/mL streptomycin (Solarbio). The viability of PBMCs was determined by trypan blue exclusion, and the cell density was adjusted to 1 × 10^6^/mL.

### Lymphocyte proliferation assay

Lymphocytes were loaded into 96-well cell culture plates with 2.0 × 10^5^ cells per well in triplicate and stimulated with media alone, 10 μg/mL S1 protein, or 10 μg/mL concanavalin A (ConA) (Solarbio). A blank control group was set up. The cells were cultured for 72 h at 37 °C with 5% CO_2_, after which 10 μL of CCK8 (Biosharp, China) was added to each well at 37 °C for 3 h, after which the absorbance was measured at 450 nm via a multimode microplate reader (Biotek Synergy HTX). The results are expressed in the form of a stimulation index (SI), which is calculated from the mean of triplicate values via the following formula:

$${\mathrm{SI}} = \frac{{{\mathrm{OD}}\,\left( {{\mathrm{samples}}} \right) - {\mathrm{OD}}\,\left( {{\mathrm{blank}}} \right)}}{{{\mathrm{OD}}\,\left( {{\mathrm{control}}} \right) - {\mathrm{OD}}\,\left( {{\mathrm{blank}}} \right)}}$$

### RT‒PCR

The serum, nasal mucus and fecal samples at 0, 1, 3, 5, 7, 10 and 14 dpc were collected for RT‒PCR detection. An RNA Minibest Universal RNA Extraction Kit (Takara Bio, Shiga, Japan) and a reverse transcription kit (Takara Bio) were used to purify the RNA and prepare the cDNA. The PEDV M protein-encoding gene was amplified via PCR via specific primers (U-PEDV M292: 5′-AATAGCATTCGGTTGTGG-3′) and L-PEDV M618: 5′-GTAGTCGCCGTGTTTAGA-3′), 1.5% agarose gel electrophoresis was used to analyse the amplification results, and the number of positive samples was counted.

### Histopathological examination of small intestinal villi

To illustrate the effects of oral inoculation of S1-CS NAs on the intestinal mucosa morphology of piglets, paraffin Sects (5 µm) of the duodenum, ileum and jejunum of piglets were prepared and stained with haematoxylin–eosin (H&E) for routine histological examination via light microscopy (Olympus, Tokyo, Japan). Villus height (VH) and crypt depth (CD) were measured, and the ratio of villus height to crypt depth (V/C) was calculated. Changes in intestinal villi were analysed for each sample on average from 5 fields.

To further evaluate the intestinal mucosal immune responses induced by oral vaccination with S1-CS NPs, IgA antibody-secreting cells in the duodenum, jejunum and ileum mucosa were analysed via immunohistochemistry. After antigen renaturation and peroxidase removal, the tissue sections were blocked for 30 min at RT with 3% BSA-PBS and then incubated with an HRP-conjugated goat anti-pig IgA antibody (1:200, Abcam, Britain) diluted in 3% BSA-PBS at 4 °C for 12 h. After rinsing 3 times with PBS, the DAB substrate chromogen (Boster Bio Tech, China) was used for development for 5 min. The number of positive cells stained with dark brown, IgA antibody-secreting cells (ASCs) in the lamina propria was counted via light microscopy (Olympus) after counterstaining with haematoxylin, dehydration and sealing with neutral balsam (Solarbio). Five fields were counted for each sample, and the average was used for analysis.

### Statistical analysis

All the data presented in this study are expressed as the mean ± standard deviation (SD) and were analysed via SPSS Statistics 19.0 software (IBM, Chicago, USA). Statistical analysis was conducted via one-way ANOVA, followed by Duncan’s multiple comparisons test. Statistical significance was considered and expressed “*” as *P* < 0.05 and “**” as *P* < 0.01.

## Results

### Preparation of S1-CS NPs

**Figure 2:**
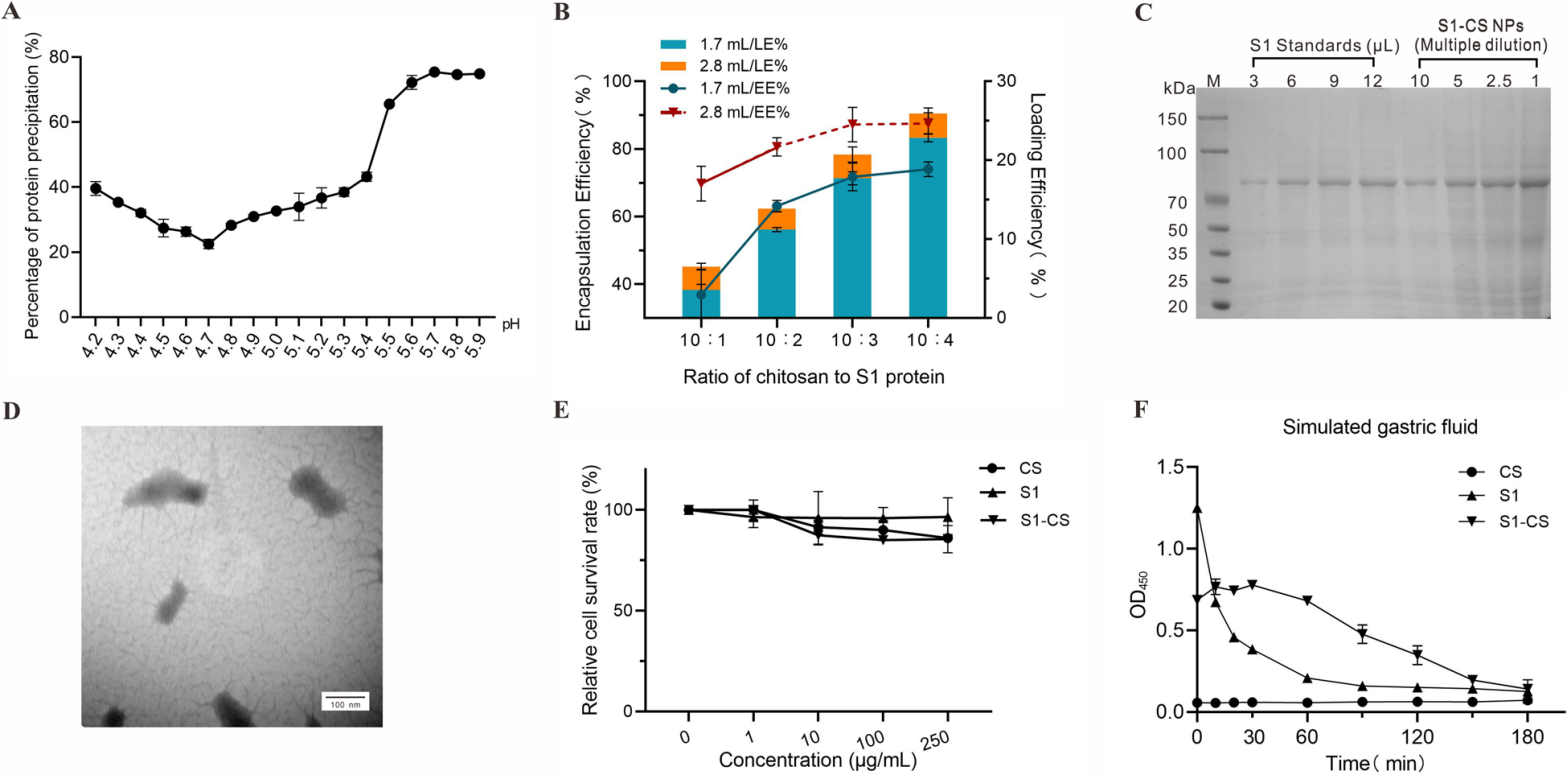
**Preparation and characterization of S1-CS nanoparticles**. **A** Effect of the pH of the chitosan solution on the solubility of the PEDV S1 protein. **B** Effects of the ratio of chitosan to S1 protein on the encapsulation efficiency (EE) and loading efficiency (LE) of S1-CS NPs. The dotted line represents the precipitation. **C** SDS‒PAGE analysis of the antigen content in S1-CS NPs. **D** TEM image of S1-CS NPs at 200 000 × magnification. **E** Cell viability evaluation of S1-CS NPs. As determined by the CCK-8 assay after 24 h. **F** Stability analysis of S1-CS in the simulated gastric fluid. CS, free S1 protein and S1-CS NPs were incubated with simulated gastric fluid (pH 1.2). All the data are presented as the mean ± SD.

On the basis of the charge characteristics of the protein, to achieve better mixing of the positively charged chitosan solution with the PEDV S1 protein (PI = 5.2) and prevent precipitation, we optimized the pH of the chitosan solution, as illustrated in Figure 2A. When the pH value of the 0.1% chitosan solution was 4.7, the least amount of precipitate was observed after mixing chitosan with S1 protein, which indicated that pH 4.7 was the optimal choice, while any increase or decrease in the pH value would have an adverse effect. When S1-CS NPs were prepared according to a ratio of chitosan to TPP of 10:1.7, as the ratio of chitosan to S1 protein increased, the EE gradually increased, reaching average values of 36.84%, 63.12%, 71.74%, and 74.02%, respectively (green line in Figure 2B). When the chitosan to TPP ratio was increased to 10:2.8, the EE progressively increased to 69.7%, 80.6%, 87.2%, and 87.5%, respectively (red line). Nevertheless, 87.2% and 87.5% EE resulted in excessive cross-linking, as evidenced by visible precipitation (as indicated by the red dashed line in Figure 2B). The optimal ratio for the preparation of S1-CS NPs was 10:2:2.8 for chitosan, S1 protein, and TPP, respectively. Moreover, the results suggested that the S1 protein potentially interferes with the cross-linking reaction between chitosan and TPP.

SDS‒PAGE was used to analyse the S1 protein in the prepared S1-CS NPs. Following electrophoresis, the band grayscales of standard S1 protein and S1-CS NPs were analysed first with ImageJ processing software (Figure 2C). The calculated content of S1 protein in the S1-CS NPs was 7538 μg/mL according to the standard curve generated from the standard S1 protein. Furthermore, TEM images of the S1-CS NPs are shown in Figure 2D. The S1-CS NPs have a diameter of approximately 80–150 nm, which is similar to the diameter of PEDV (95–190 nm), and exhibit an irregular elliptical shape.

### Non-cytotoxicity of S1-CS NPs

The cytotoxicity of S1-CS NPs was evaluated by analysing the relative cell viability after incubation with PK-15 cells. The trend of the change in relative cell viability was similar to that of the coincubation of chitosan or S1-CS NPs with PK-15 cells. S1 protein and S1-CS NPs had no significant effect on relative cell viability (*P* > 0.05), despite a slight decline in relative cell viability when the concentration of chitosan was greater than 10 μg/mL (shown in Figure 2E).

### S1-CS NPs resist the digestion of simulated gastric fluid

After co-incubation of S1-CS NPs or S1 protein with simulated gastric fluid, the level of S1 protein in the supernatant was determined to evaluate the protective effect of chitosan on the S1 protein. As shown in Figure 2F, the free S1 protein rapidly degraded in the simulated gastric fluid, with 30.7% of the S1 protein remaining at 30 min and 88.2% remaining after 90 min. In contrast to the free S1 protein, the S1 protein was released slowly from the S1-CS NPs in the simulated gastric fluid, with 54.3% and 27.9% of the S1 protein remaining at 60 min and 120 min, respectively. The results revealed that S1-CS NPs were able to withstand gastric fluid digestion to gradually release the S1 protein, which is conducive to the continuous induction of the immune response.

### S1-CS NPs induce systemic and intestinal mucosal immune responses in mice

**Figure 3:**
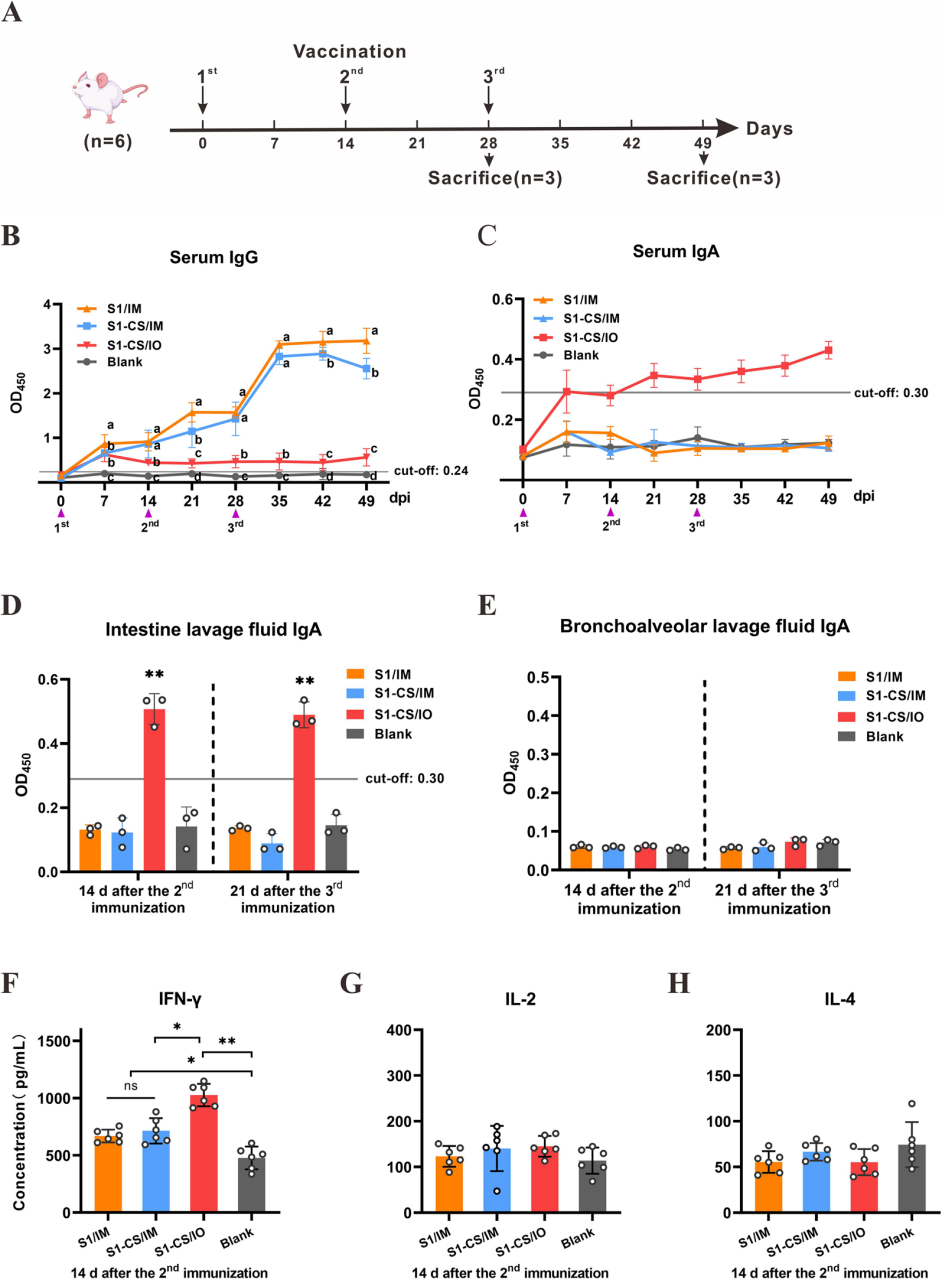
**Experimental timeline of the use of specific antibodies and cytokines in mice. A** Schedule of immunization and sample collection in mice. Blood samples were collected from the tail vein of the mice weekly before and after immunization. At 14 days after the second immunization and 21 days after the third immunization, the intestine and lung airway lavage fluid were collected. **B** Dynamics of serum-specific IgG. **C** Dynamics of serum-specific IgA. **D** and **E** Specific mucosal IgA in small intestinal lavage fluid and bronchoalveolar lavage fluid. **F**–**H** Levels of IFN-γ, IL-2 and IL-4 in the serum at 14 days after the second immunization.

To evaluate the immunogenicity of S1-CS NPs, mice were inoculated intramuscularly or orally with S1-CS NPs according to the experimental procedure (Figure 3A). As shown in Figure 3B, specific IgG antibodies were detected in two intramuscular immunization groups (S1/IM and S1-CS/IM) and the oral immunization group (S1-CS/IO) on day 7 after primary immunization. The antibody concentration gradually increased in the S1/IM and S1-CS/IM groups but not in the S1-CS/IO group. The average antibody levels of the S1/IM and S1-CS/IM groups were significantly higher than those of the S1-CS/IO group 14 days after primary immunization (*P* < 0.05). Specifically, the IgG antibody level in the S1-CS NP oral inoculation group, although at a lower level, was still higher than that in the blank group (*P* < 0.05).

Only the serum IgA level in the S1-CS/IO group (*P* < 0.05) significantly increased at 7 days after the first immunization (Figure 3C). There was no significant difference between the S1/IM, S1-CS/IM and blank groups (*P* > 0.05). Similarly, markedly elevated mucosal IgA was present in the intestinal mucosa of the S1-CS/IO group at 14 days after the second immunization and 21 days after the third immunization (*P* < 0.01). No specific mucosal IgA was detected in the other groups (Figure 3D). In the bronchoalveolar lavage fluid of all the groups, specific mucosal IgA was not detected (Figure 3E). These results indicate that S1-CS NPs stimulate not only the systemic immune response but also the local intestinal rather than the respiratory mucosal-specific IgA response following oral immunization.

To further characterize the cytokines induced by the S1-CS NPs, Th1 cytokines (IFN-γ and IL-2) and Th2 cytokines (IL-4) in the serum were analysed via ELISA on day 14 after the second immunization. Compared with that in the blank group, IFN-γ in the S1/IM, S1-CS/IM and S1-CS/IO groups increased (*P* < 0.05), and there was greater IFN-γ upregulation in the S1-CS/IO group than in the S1/IM or S1-CS/IM groups (*P* < 0.01) (Figure 3F). IL-2 and IL-4 levels were not significantly different among the groups (*P* > 0.05) (Figure 3G and H). These findings suggest that the S1-CS NPs promoted the IFN-γ response, especially when they were inoculated orally.

### Oral administration of S1-CS NPs protects piglets

**Figure 4:**
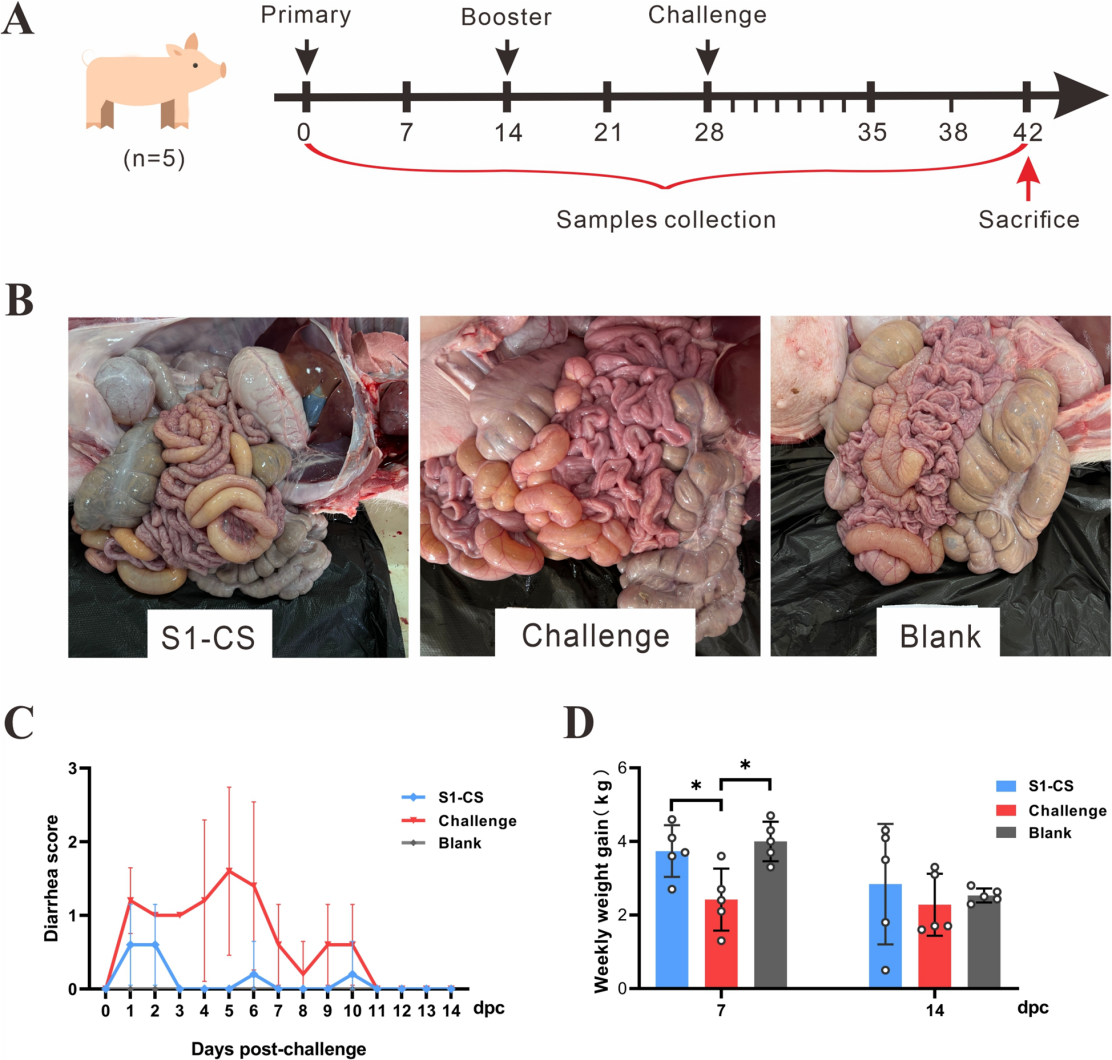
**Experimental timeline, gross lesions, faecal consistency score and average weekly weight gain in piglets**. **A** Schedule of immunization, challenge, sacrifice and sample collection in piglets. **B** Gross lesions in the small intestine of piglets at 14 days post-challenge. **C** Mean faecal consistency score in piglets post-challenge. The fecal consistency was scored as follows: 0, solid; 1, pasty; 2, semiliquid; and 3, liquid. **D** Average weekly weight gain post-challenge.

To evaluate the protective efficacy of oral S1-CS NPs on piglets, weekly weight gains were measured, and faecal consistency was scored after challenge. One piglet in the S1-CS group had pasty stool only at 1 and 2 dpc. One piglet in the challenge group developed watery diarrhea for 4 days from 4 dpc, and the remaining piglets had pasty stool until 10 dpc. The mean faecal consistency scores of the S1-CS NPs and challenge groups were not significantly different (*P* > 0.05), but they were greater than those of the blank group (Figure 4C). After 14 days of challenge, the small intestines of infected pigs were obviously distended and filled with yellowish fluid, and the small intestinal mucosa was congested, while there were almost no lesions in the S1-CS NP group, similar to those in the blank group (Figure 4B). One week after challenge, the average weekly gain of piglets decreased significantly only in the challenge group (*P* < 0.05), while there was no significant difference between the S1-CS NP group and the blank group (*P* > 0.05) (Figure 4D). At 14 days after the challenge, there was no significant difference in weight gain among the groups.

### Oral administration of S1-CS NPs induces systematic and intestinal mucosal antibody responses in piglets

**Figure 5:**
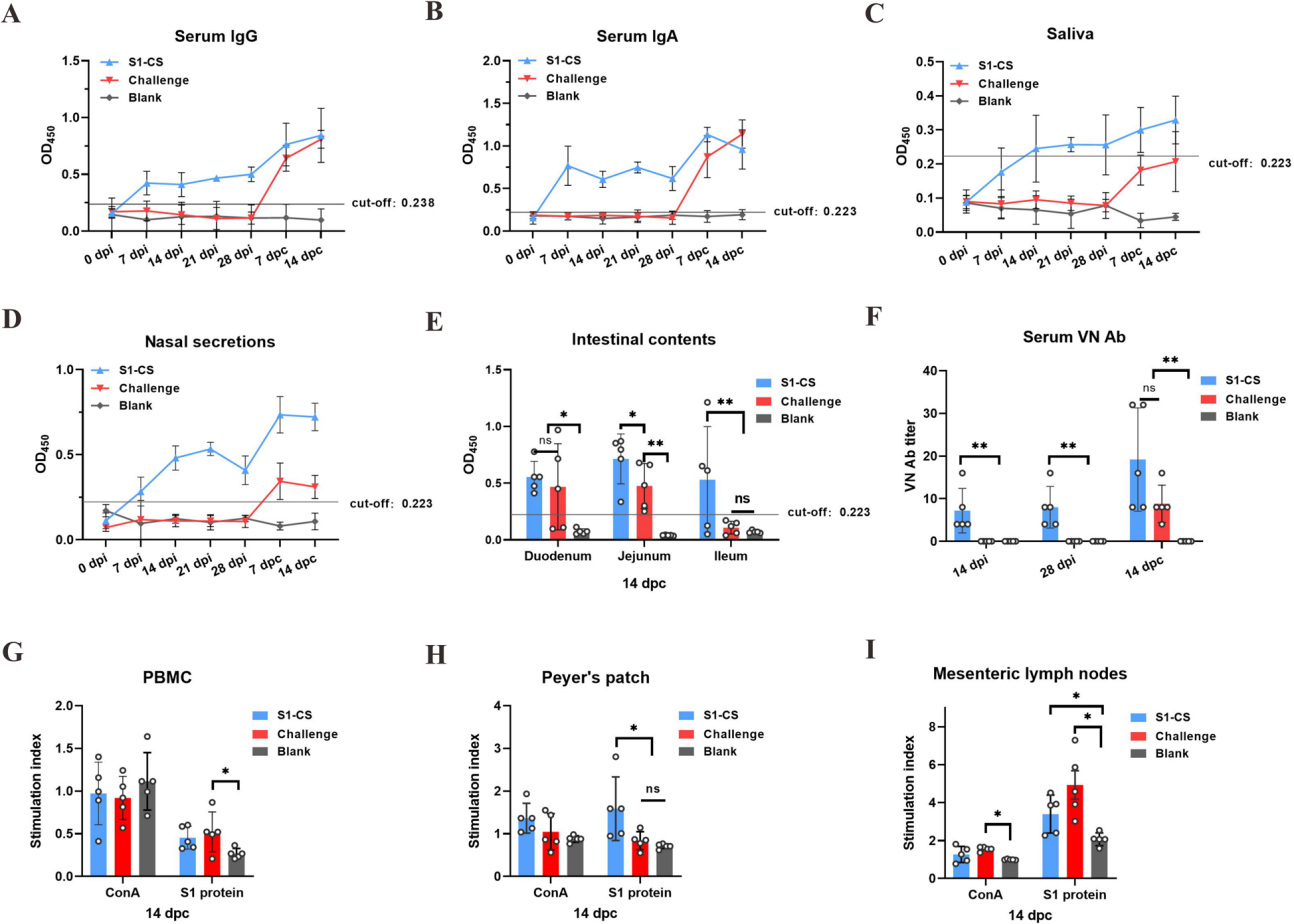
**Specific antibodies and lymphocyte proliferative activity in piglets**. **A**, **B** Dynamics of specific IgG and IgA in serum. **C**, **D** Dynamics of local specific mucosal IgA antibodies in saliva and nasal secretions, respectively. **E** Specific mucosal IgA antibody titres in the small intestine contents of piglets post-challenge. **F** Virus-neutralizing antibody titre in the serum of piglets in each group. **G**–**I** Stimulation indices of PBMCs and lymphocytes in PPs and MLNs at 14 days after challenge of piglets.

Systemic and mucosal antibody (IgG and IgA) and serum neutralizing antibody responses were evaluated after piglets were orally vaccinated with S1-CS NPs following the experimental procedure shown in Figure 4A. Serum-specific IgG and IgA were positive on day 7 after primary immunization but remained at relatively low levels (Figures 5A and B). After challenge, the IgG and IgA levels further increased in the S1-CS group but were not significantly different from those in the challenge group (*P* > 0.05). Both groups presented significantly greater antibody responses than did the blank group post-challenge (*P* < 0.05).

In terms of mucosal antibodies, specific mucosal IgA antibody responses in the saliva (Figure 5C) and nasal mucus (Figure 5D) of piglets were significantly greater in the S1-CS group than in the challenge and blank groups after primary immunization (*P* < 0.05). Salivary mucosal IgA levels surpassed the cut-off of 0.223 on day 14 after the first immunization and remained low thereafter. Nasal mucosal IgA levels peaked (0.534 ± 0.039) on day 7 after the second immunization and then began to decrease. After challenge, the specific mucosal IgA antibody response in saliva and nasal mucus significantly increased in the S1-CS group (*P* < 0.05). There was a trend toward salivary mucosal IgA but no positive conversion in the challenge group. Moreover, at 14 days post-challenge, specific mucosal IgA in the piglet duodenal, jejunal, and ileal contents (Figure 5E) of the S1-CS group markedly increased (*P* < 0.05), as did the duodenal and jejunal contents of the challenge group (*P* < 0.05). The jejunal and ileal mucosal IgA responses of the S1-CS group were significantly greater than those of the challenge group.

The serum neutralizing antibody titres of the S1-CS group were 1:7.2 and 1:8.0 at 14 days after the primary and secondary immunizations, respectively (Figure 5F). After challenge, the titre reached 1:19.2 but was not significantly different from the titre (1:8.8) of the challenge group (*P* > 0.05). The above results indicate that oral inoculation with S1-CS NPs effectively induced systemic IgG and IgA and intestinal mucosal IgA antibodies, as well as a neutralizing antibody immune response in piglets.

### Specific proliferation activity of lymphocytes increases in Peyer’s patches and mesenteric lymph nodes of piglets

To determine the effects of oral immunization with S1-CS NPs on lymphocyte proliferative activity, lymphocytes were prepared from peripheral blood, PPs, and MLNs at 14 dpc. In both the Con A and S1 groups, S1-CS NP inoculation failed to contribute to an increase in the SI of PBMCs compared with that of the challenge group and the blank group (*P* > 0.05) (Figure 5G). However, the lymphocyte SI clearly increased in PPs and MLNs stimulated with the S1 protein rather than with Con A between the S1-CS group and the blank group (*P* < 0.05) (Figure 5H–I). In PPs, the SI of the S1-CS group was also significantly greater than that of the challenge group (*P* < 0.05) (Figure 5H). The results showed that oral inoculation with S1-CS NPs induced a population of S1-responsive lymphocytes in gut-associated lymphoid tissue (GALT).

### Oral administration of S1-CS NPs reduces the rates of virus shedding and viremia in piglets

To detect PEDV shedding from the rectum and nasal cavity, as well as viremia, viral nucleic acid in faeces, nose secretions, and serum was detected by RT‒PCR after challenge. No PEDV nucleic acid was detected in the faeces, nasal secretions or serum of the control piglets before or after challenge. PEDV shedding began from the feces and nasal secretions of piglets in the challenge group as early as 1 dpc and persisted until 14 dpc. In the S1-CS group, virus shedding occurred in the feces at 1 dpc and in the serum at 3 dpc, but not in the nasal secretions, and it persisted until day 14 after challenge (Table 1). Notably, the total positive rates of virus detection in faeces, nasal secretions and serum were lower than those in the challenge group. These results indicated that oral inoculation of S1-CS nanoparticles effectively reduced PEDV shedding and viremia.

**Table 1:** **Dynamics of viral shedding and viremia post-challenge measured by RT‒PCR**

Groups	Days post-challenge (d)								Total positive rates
		0	1	3	5	7	10	14	
S1-CS	Feces	0/5^a^	1/5	1/5	0/5	2/5	3/5	2/5	30.0%
	Nasal secretion	0/5	0/5	1/5	0/5	2/5	2/5	2/5	23.3%
	Serum	0/5		1/5		1/5		0/5	13.3%
Challenge control	Feces	0/5	3/5	4/5	3/5	4/5	3/5	3/5	66.7%
	Nasal secretion	0/5	1/5	2/5	3/5	4/5	4/5	3/5	56.7%
	Serum	0/5		4/5		3/5		2/5	60.0%
Blank	Feces	0/5	0/5	0/5	0/5	0/5	0/5	0/5	0
	Nasal secretion	0/5	0/5	0/5	0/5	0/5	0/5	0/5	0
	Serum	0/5		0/5		0/5		0/5	0

^a^Ratio of the number of positive piglets to the total number of piglets tested.

### Oral administration of S1-CS NPs prevents damage to the small intestinal villi of piglets

**Figure 6:**
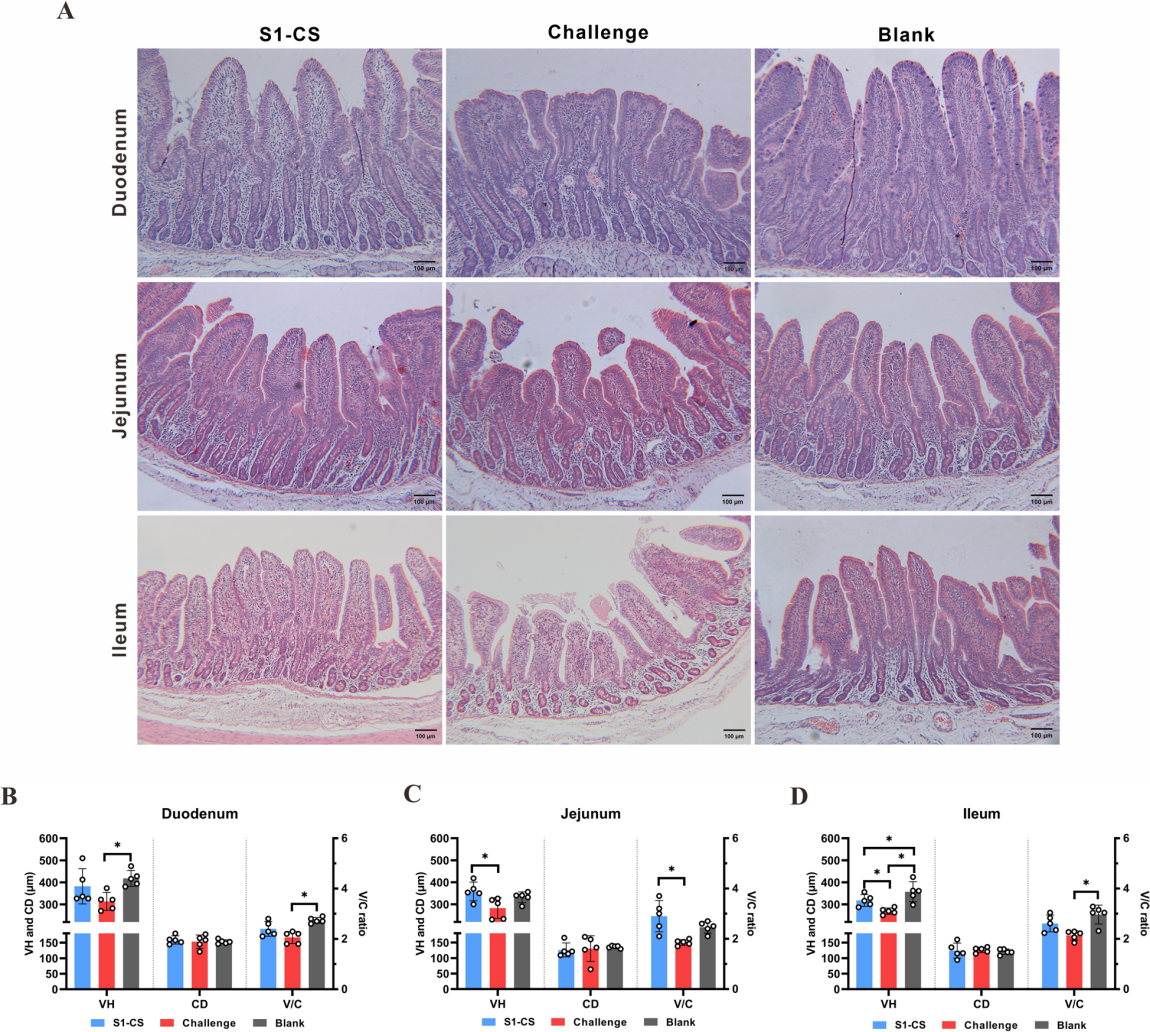
**Microscopic lesions of the small intestine, villus height and the V/C ratio 14 days after challenge (HE staining, scale = 100 μm)**. **A** Microscopic lesion of the duodenum, jejunum, and ileum. **B**–**D** Measurement and statistical analysis of villus height (VH), crypt depth (CD) and the ratio of villus height to crypt depth (V/C) in the duodenum, jejunum and ileum, respectively.

Compared with those of the blank group, the duodenal VH and V/C ratios of the challenge control group were lower (*P* < 0.05), but those of the S1-CS group were not significantly different (*P* > 0.05) (Figures 6A and B). There was no significant difference in crypt depth among the groups (*P* > 0.05). The jejunum VH and V/C ratio of the S1-CS group were significantly greater than those of the challenge control group (*P* < 0.05) and not significantly different from those of the blank group (*P* > 0.05) (Figures 6A and C). The ileal VH in the S1-CS group was lower than that in the blank group but significantly greater than that in the challenge group (*P* < 0.05) (Figures 6A and D). The V/C ratio in the S1-CS group was not significantly different from that in the blank and challenge groups (*P* > 0.05), whereas that in the challenge group was significantly lower (*P* < 0.05). There was no significant difference in CD among the groups. These results showed that oral inoculation of S1-CS NPs provided effective immune protection to piglets after PEDV challenge by improving the morphology of the intestinal villi.

### Oral administration of S1-CS NPs increases the number of IgA-secreting cells in the small intestines of piglets

**Figure 7:**
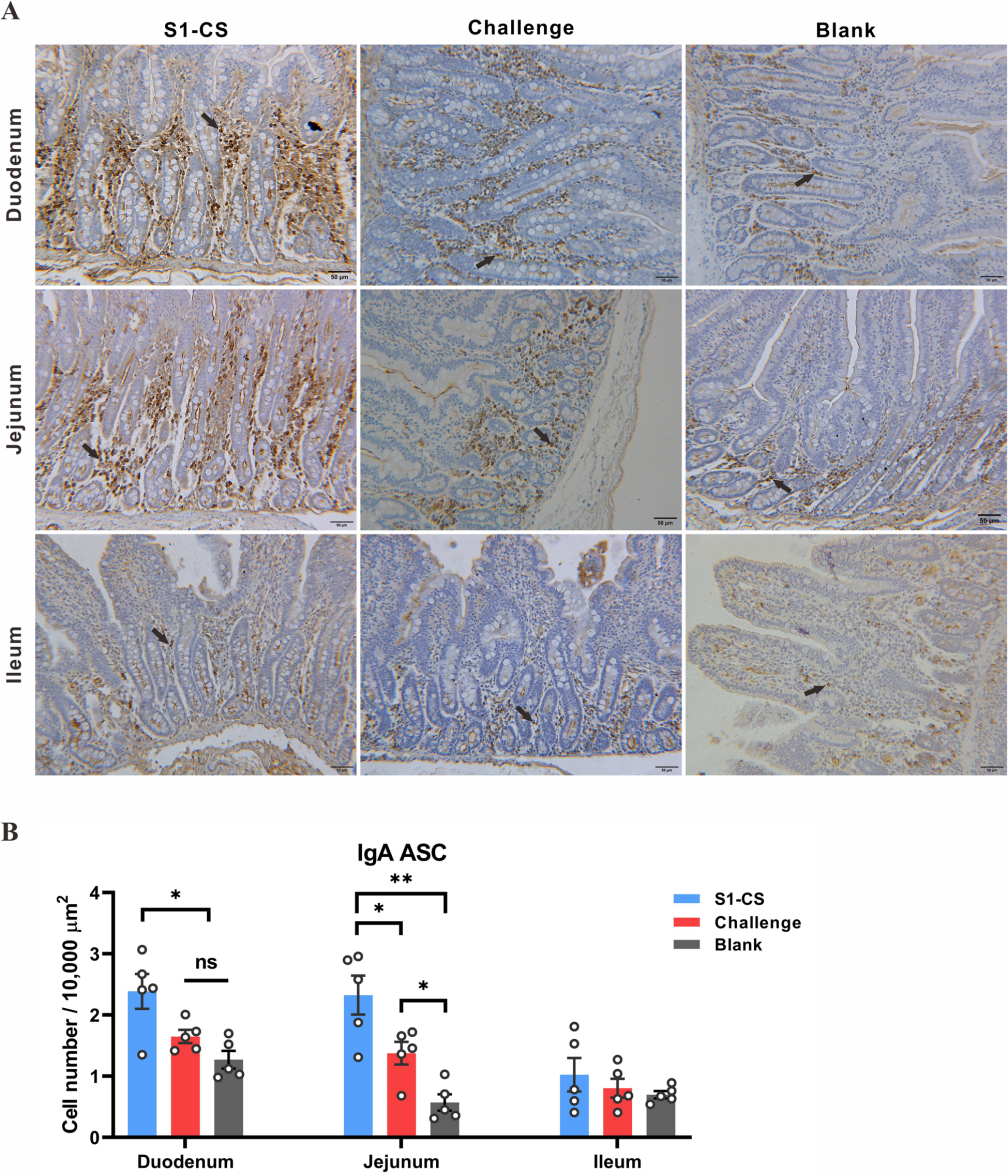
**Analysis of IgA antibody-secreting cells in the small intestine (DAB staining, scale = 50 μm)**. **A** Immunohistochemistry analysis of IgA antibody-secreting cells (ASCs, indicated by arrows) in the lamina propria of the duodenum, jejunum, and ileum. **B** Statistical analysis of ASCs in the duodenum, jejunum and ileum.

The number of IgA ASCs in the duodenum and jejunum mucosa in the S1-CS group was significantly greater than that in the challenge control group and blank group (*P* < 0.05). There was no significant difference in the number of IgA ASCs in the ileum mucosa among all the groups (*P* > 0.05) (Figures 7A and B). In conclusion, oral inoculation with S1-CS NPs induced an increase in IgA ASC in the duodenum and jejunum of piglets.

## Discussion

Intestinal mucosal immunity plays a key role in preventing PEDV infection through damage caused by the virus to the small intestinal mucosa. Vaccination is the most effective and economical method for controlling the spread of PED. However, the immunization effects of vaccines, inactivated vaccines and attenuated vaccines used in the clinic are unsatisfactory and cannot effectively control the occurrence of PED. Inactivated vaccines are usually insufficient to stimulate intestinal mucosal immunity [19]. Although modified attenuated vaccines generally show better efficacy, they may lead to virus recombination and even cause outbreaks in pigs [20]. In addition, research on attenuated vaccines that address the speed of virus strain mutation is difficult because PEDV is prone to variation. Over time, owing to the mutation of the virus and the low degree of cross-protection among different strains, the protective efficacy of the original inactivated and attenuated vaccines has weakened [21]. Thus, there is an urgent need to develop an innovative and effective mucosal vaccine without biosafety risks.

Compared with inactivated and attenuated vaccines, subunit antigens have precise antigen components and no ability to replicate infection, indicating greater biosafety characteristics [22]. However, owing to the lower immunogenicity of subunit antigens, adjuvants or antigen delivery systems are needed to increase their immunological efficiency [23]. Chitosan-based delivery systems have been regarded as potential mucosal adjuvants and antigen delivery systems. Their inherent positive surface charge enables them to bind strongly to negatively charged materials such as cell surfaces or mucus, increasing the efficiency of inducing mucosal immune responses [24]. In recent years, chitosan has been demonstrated to possess unique mucoadhesiveness and the ability to open tight junctions between epithelial cells, enhancing the transport of antigens to immune induction and effector sites [25]. Furthermore, the use of chitosan as a delivery system has advanced in many forms, including microspheres, nanoparticles, hydrogels, and emulsions [13, 25]. The nanoparticles are easily recognized and processed by APCs, and the S1-CS NPs prepared in this study have a diameter of approximately 80–150 nm, similar to the diameter (95–190 nm) of the PEDV particles, facilitating uptake by the APC. There are various methods for preparing chitosan nanoparticles, and among them, the ionic gelation method is widely used because of its mild preparation conditions and the absence of toxic solvents [26, 27]. As a nontoxic pentavalent polyanionic crosslinking agent, when TPP is mixed with chitosan, CS-NPs are instantly formed through electrostatic interactions. The particle size and antigen entrapment efficiency of CS-NPs can be regulated by controlling the ratio of sodium tripolyphosphate [28]. In the studies by Rázga et al. [29] and Nunes et al. [30], nanoparticles of approximately 400 nm and 180 nm were prepared using 0.056% (w/v) CS solution (pH 6.0) and 0.1% (w/v) CS solution (pH 5.2), respectively. In this study, considering the solubility of the S1 protein, we controlled the pH of the CS solution to approximately 4.7, resulting in S1-CS nanoparticles with a size of approximately 80–150 nm. Therefore, we speculate that the particle size of CS-NPs is correlated with the concentration and pH of the CS solution, the encapsulation material, and the TPP ratio. Furthermore, in this study, the antigen EE reached a maximum of 80.6%, which is a certain gap compared with the encapsulation rates exceeding 90% reported by Cevher et al. [31] for encapsulating the BSA protein. This difference may be related to the size of the materials being loaded. The trimeric S1 protein has a total molecular weight of approximately 237 kDa, which is 4 times greater than that of the BSA protein (66 kDa). On this basis, we infer that the encapsulation efficiency increases as the molecular weight of the loaded materials decreases. However, no direct evidence has been found to support the correlation between the size of the materials and the chitosan encapsulation rate.

To ensure that the antigen can induce an effective immune response, the design of oral vaccines must consider the destructive effects of harsh gastrointestinal environment factors such as pH and digestive enzymes on antigens [13]. Chitosan nanoparticles exhibit resistance to digestion in vitro and a certain degree of sustained release capability [32]. In the simulated gastric fluid digestion test during the first 2 h, the release ratio of the encapsulated chitosan was only 41.55%, which was significantly lower than that of the control group (76.9%). In another study [33], with chitosan nanoparticles loaded with proanthocyanidin, the release rate within the first hour reached 49.6%. In a simulated digestion experiment in vitro, we observed a similar effect on the sustained release of S1 protein by S1-CS NPs, which may be associated with insufficient exposure of the S1 protein encapsulated within the sponge-like structure of chitosan.

Immunological experiments in mice revealed that different inoculation routes significantly affected the immune characteristics of S1-CS NPs. While intramuscular injection of S1-CS NPs effectively triggered the systemic immune response, it struggled to elicit an antigen-specific immune response on mucosal surfaces, which aligns with the results reported by Lycke [34]. In contrast, oral inoculation of S1-CS NPs, while capable of inducing the production of IgA in mouse serum and local mucosal IgA in the intestines, has limited efficacy in eliciting systemic IgG antibody responses, which is consistent with the findings of Wu [35]. In addition, this result also verified the resistance of S1-CS NPs to gastric fluid digestion. Most notably, no specific IgA was detected in the respiratory mucosa of all groups of mice, which contradicts the findings of Ding et al. [36] and Zhuo et al. [37] in their research on influenza A and SARS-CoV-2 viruses, which may be attributed to the antigen characteristics and tissue tropism of the PEDV-S protein [16].

Cytokines play crucial roles in maintaining the balance between Th1 and Th2 immune responses and in the defense against infectious diseases [38]. This study revealed that the oral administration of S1-CS NPs significantly increased the level of the Th1-type cytokine IFN-γ, whereas that of IL-4 was unchanged in mice. These results suggested that chitosan could stimulate a Th1-type immune response. The activation of DCs by chitosan nanoparticles likely results from their cationic surface properties and mucoadhesive capacity, which collectively enhance targeted delivery to DCs and potentiate cellular immune responses [13, 39, 40].

Immunological protection experiments confirmed that oral inoculation of piglets with S1-CS nanoparticles induced systemic immunity and mucosal immunity such that the piglets achieved immune protection, which was based on a reduction in clinical signs, intestinal pathological changes, virus shedding, and viremia. Neutralizing antibodies play a crucial role in eliminating viruses and reducing viremia or shortening the duration of viremia [41]. In this study, low levels of serum neutralizing antibodies were detected after oral inoculation of S1-CS NPs and challenge, which may be one reason for the low positive rate of viremia and the short duration of viremia in immunized piglets.

The intestinal immune system can be divided into inductive sites and effector sites [42]. The inductive sites primarily comprise gut-associated lymphoid tissue, such as PPs and isolated lymphoid follicles (ILFs), as well as MLNs. The effector sites are located in the epithelium and lamina propria of the small intestine, where they contain many activated T cells and antibody-secreting plasma cells [43, 44]. The lymphocytes in the PPs and MLNs in the S1-CS group exhibited significantly greater proliferative activity upon specific stimulation in the present study. Furthermore, many IgA ASCs in the lamina propria of the duodenum and jejunum appeared in piglets orally administered S1-CS NPs. This may be related to the presence of mucosal IgA in secretions from the oral and nasal cavities, the contents of the intestine, and the presence of IgA in the serum [45]. However, no similar changes were observed in the ileum, which may be due to the degradation of S1-CS before it reaches the ileum.

The enhanced intestinal mucosal immunity induced by S1-CS can be attributed to two main factors: one is the inherent adsorption capacity of chitosan, which may play an auxiliary role, and the other is antigen targeting to cellular receptors, such as pAPN, in the gut, which significantly enhances mucosal immunogenicity. A previous study showed that F4ac fimbriae targeting the pAPN receptor can promote both systemic and mucosal antibody responses [5]. Furthermore, microparticles functionalized with monoclonal antibodies or heavy-chain-only antibodies against pAPN also increase their uptake by intestinal epithelial cells and elicit systemic and mucosal immune responses [46]. The above findings indicate that the PEDV S1 protein, as an oral antigen, intrinsically enhances mucosal immunity through receptor-specific targeting.

PEDV mainly infects small intestinal epithelial cells and leads to diarrhea, atrophy and shedding of intestinal villi, as well as deepening of crypts, whereas the height of small intestinal villi and the V/C ratio are positively correlated with nutrient absorption capacity and growth performance [47, 48]. In this study, the oral administration of S1-CS NPs increased the height of the villi in the small intestine and the V/C ratio to some extent, which may be related to the protective effect.

In conclusion, this study combined a chitosan nanoparticle delivery system with subunit antigens to design the chitosan-based nanoparticle PEDV S1-CS NPs. S1-CS NPs, with nanoparticle sizes ranging from approximately 80 to 150 nm, exhibited no cytotoxicity and resisted gastric fluid digestion in vitro. Oral immunization of piglets with S1-CS NPs has the ability to induce specific IgA antibody responses in both the systemic and intestinal mucosa; increase the number of ASCs in the small intestine; and reduce clinical signs, intestinal pathological changes, viremia and virus shedding, which demonstrates that S1-CS NPs have the potential to be developed as oral PEDV mucosal vaccines.

## Data Availability

The datasets used in the current study are available from the corresponding author upon reasonable request.
